# Bhlhe40/Sirt1 Axis-Regulated Mitophagy Is Implicated in All-*Trans* Retinoic Acid-Induced Spina Bifida Aperta

**DOI:** 10.3389/fcell.2021.644346

**Published:** 2021-04-27

**Authors:** Lu Zhao, Dan Liu, Wei Ma, Hui Gu, Xiaowei Wei, Wenting Luo, Zhengwei Yuan

**Affiliations:** Key Laboratory of Health Ministry for Congenital Malformation, Shengjing Hospital, China Medical University, Shenyang, China

**Keywords:** spina bifida aperta, mitophagy, Bhlhe40, Sirt1, autophagy, resveratrol

## Abstract

Neural tube defects (NTDs) are the most severe congenital malformations that result from failure of neural tube closure during early embryonic development, and the underlying molecular mechanisms remain elusive. Mitophagy is the best-known way of mitochondrial quality control. However, the role and regulation of mitophagy in NTDs have not yet been elucidated. In this study, we used an all-*trans* retinoic acid (ATRA)-induced rat model to investigate mitophagy and its underlying mechanism in spina bifida aperta (SBA). The results of western blot, immunofluorescence and RT-qPCR analyses indicated that mitophagy was impaired and Sirt1 was downregulated in SBA. Administration of resveratrol-a strong specific Sirt1 activator-activated Sirt1, thus attenuating autophagy suppression and ameliorating SBA. RNA-sequencing and bioinformatics analysis results indicated that transcriptional regulation played an important role in NTDs. A luciferase reporter assay was performed to demonstrate that the transcription factor Bhlhe40 directly bound to and negatively regulated *Sirt1* expression. Further, we discovered that the Bhlhe40/Sirt1 axis regulated mitophagy in neural stem cells. Collectively, our results for the first time demonstrate that Bhlhe40/Sirt1 axis regulated mitophagy is implicated in ATRA-induced SBA. Our findings provide new insights into pathogenesis of NTDs and a basis for potential therapeutic targets for NTDs.

## Introduction

Neural tube defects (NTDs), such as anencephaly and spina bifida, represent the most severe and common congenital malformations of the central nervous system resulting in infant deaths and severe disabilities, bring undue burden to the ill infant and their families as well as society (Greene and Copp, 2014). The global incidence of NTDs is approximately 18.6 in 10,000 births globally (Blencowe et al., 2018). NTDs have a complex etiology and mainly result from incomplete closure of the neural tube during early embryonic development. Failure of neural folding or closure of neuropores is caused by an interaction of genetic and environmental factors, lack of nutrients or other factors (Bhandari and Thada, 2020). Current treatment strategies for NTDs are not satisfactory because the pathogenesis is insufficiently understood (Norkett et al., 2016; Radic et al., 2019). In-depth study of the mechanisms underlying NTD pathogenesis and the development of scientific prevention and treatment methods are of great significance for reducing the malformation and disability rates, thus improving patient quality of life and reducing societal burden.

Neural tube defects are considered polygenic genetic diseases. Extensive studies of vertebrate models have partially elucidated the mechanism of neural tube closure and its failure during NTDs. Studies in various animal models have indicated that the pathogenesis of NTDs involves multiple molecular pathways (Juriloff and Harris, 2018). Neural fold bending, elevation, and fusion is regulated via various molecular events, including the non-canonical wnt/planar cell polarity pathway, sonic hedgehog/bone morphogenetic protein signaling, and several transcription factors regulation (Nikolopoulou et al., 2017; Wang et al., 2019). Recently, it has been found that the dysregulation of apoptosis and autophagy in fetal central nervous tissues is associated with NTD development. Mutations in apoptosis-related genes *CASP3*, *CASP9*, and *APAF1* reportedly are conductive to human NTDs (Zhou et al., 2018). In another recent study, deletion of the forkhead box O3a transcription factor gene eliminated the inhibitory effect of maternal diabetes on the expression of autophagy-related protein and thus alleviated diabetes-induced cell apoptosis and NTDs in a mouse model (Xu et al., 2019). Further research is still warranted to reveal the mechanisms behind NTDs.

Mitochondria are essential organelles that provide cells with energy and buffering cytoplasmic calcium. In addition, they are the sites of harmful reactive oxygen species (ROS) production and secrete pro-apoptotic factors (Giorgi et al., 2012). A growing body of evidences suggested that neurodevelopment requires well-structured mitochondria to maintain nervous system function (Khacho and Slack, 2018). Notably, mitochondrial damage recently has been proven to be associated with NTDs (Chen X. et al., 2017). Maternal diabetes can significantly increase the number of defective mitochondria in the neuroepithelia of mouse embryos, but does not affect the total number of mitochondria (Xu et al., 2013). Our previous study revealed that compared with normal rat embryos, mitochondrial respiratory efficiency and the adenosine triphosphate content were decreased in rat embryos with all-*trans* retinoic acid (ATRA)-induced spina bifida aperta (SBA), indicating that mitochondrial dysfunction is associated with ATRA -induced SBA in rat (Xue et al., 2018).

Autophagy is a major cellular pathway that facilitates the elimination of the toxic proteins and damaged organelles by lysosomal machinery. Previous studies have identified a linear association between autophagy dysfunction and NTDs (Xu et al., 2019; Ye et al., 2020). Mitophagy is a selective form of autophagy that mediates the elimination of defective mitochondria and is the only known way to selectively remove entire mitochondria (Lou et al., 2020) and control mitochondrial quality (Liu et al., 2014). Promotion of mitophagy could alleviated the development of neurological deficits due to traumatic brain injury in mice (Lin et al., 2020). Autophagy inducer rapamycin promoted mitophagy and exerted neuroprotective effects in spinal cord ischemia-reperfusion injury mouse model (Li et al., 2018). Therefore, we hypothesized that mitophagy may play an important role in the pathogenesis of NTDs and elucidating its role and underlying molecular mechanisms may reveal potential therapeutic targets.

Master transcription factors regulate autophagy and lysosomal gene expression by coordinating the expansion of autophagy-lysosomal fitness for mitophagy (Fang et al., 2016; Tan and Wong, 2017). These transcription factors include transcription factor EB and forkhead box O, which are activated by silent information regulator factor 2-related enzyme 1 (Sirt1) (Brunet et al., 2004; Ivankovic et al., 2016). Sirt1, best-studied member of the sirtuin family, is a critical regulator of autophagy (Tang, 2016). Recently, studies have shown that Sirt1 is vital for the disposal of defective mitochondria by mitophagy (Fang, 2019; Wang et al., 2020). In mice, environmental stresses reportedly can activate Sirt1 and lead to NTD-like phenotypes (Li et al., 2017). However, a microarray study demonstrated that Sirt1 was downregulated in a valproic acid-induced NTD mouse model (Okada et al., 2005). Further, Sirt1 was decreased in embryonic neural stem cells in diabetic pregnancy due to upregulation of its regulator, miRNA-30b (Ramya et al., 2017). Given these inconsistent results, more in-depth research is urgently needed to elucidate the role of Sirt1 in NTDs.

In the present study, we used an ATRA-induced SBA rat model to investigate mitophagy and Sirt1 expression. RNA-sequencing (RNA-seq) and bioinformatics analysis were performed to find the underlying molecular regulatory mechanism in SBA. Neural stem cells were used to verify the mitophagy and its regulatory mechanism. The aim of our study is to offer new insights into the pathogenesis underlying NTDs.

## Materials and Methods

### Experimental Animals and Treatments

Outbred female Wistar rats (240–300 g) were obtained from the Animal Center of Shengjing Hospital associated with China Medical University (Shenyang, China) at 10–12 weeks of age. On embryonic day (E) 10, pregnant rats were given a single intragastric dose of ATRA (4% [wt/vol] in olive oil; 140 mg/kg body weight) (Sigma-Aldrich, United States) or control treatment (olive oil only). Resveratrol (RSV; *trans*-3,5,4′-trihydroxystilbene) (MedChemExpress, NJ, United States; 40 mg/kg body weight), suspended in 50% saline, 5% DMSO, 5% Tween80, and 40% PEG400, was injected intraperitoneally once a day from E8 to E17. From E11 to E19, the posterior spinal cords of embryos were isolated, immediately snap-frozen, and stored at −80 °C until use for detecting gene and protein expression detection. Specimens for histological analysis were isolated, then fixed in 4% paraformaldehyde for 24 h. All animal experiments were approved by the Medical Ethics Committee of Shengjing Hospital of China Medical University.

### Cell Culture and Treatments

Mouse C17.2 neural stem cells were maintained in minimum essential medium (Gibco-Invitrogen, Carlsbad, CA, United States) supplemented with 10% fetal bovine serum (Gibco), 1% penicillin/streptomycin (100 U/mL and 100 μg/mL, respectively; Life Technologies), and 1% minimum essential medium non-essential amino acids (Gibco) in a humidified environment at 37°C with 5% CO_2_.

C17.2 cells were seeded in 6-well plates (1 × 10^5^ cells/well) and cultured before transfection. Plasmids pcDNA3.1(+)-CMV-Bhlhe40 and pcDNA3.1(+)-CMV-MCS were purchased from SyngenTech (Beijing, China). The cells were transfected with 1.5 μg plasmids or 100 pmol small interfering (si)RNAs in 2 mL of culture medium per well, using LipoFiter^TM^ liposomal transfection reagent (Hanbio Technology, China). After transfection for 48 h, the cells were harvested.

For Sirt1 activation or inhibition, RSV (a specific activator of Sirt1; 5 μM in DMSO) or EX527 (a specific inhibitor of Sirt1; 5 μM in DMSO; MedChemExpress) was added to the cell culture for 12 h.

### Transmission Electron Microscopy (TEM)

Mitochondrial structures were examined by TEM. Briefly, embryonic lumbo-sacral neural tube on E12 were collected carefully, immediately fixed in 2.5% glutaraldehyde, and post-fixed in 1% osmium tetraoxide. Neuroepithelia were cut and viewed under a H-7650 transmission electron microscope (Hitachi, Japan) operated at 80 KV.

### Mitochondrial Isolation

Minute^TM^ mitochondrial extraction kits (Invent Biotechnologies, Inc., Plymouth, MN, United States) were used for mitochondrial isolation of C17.2 cells according to the manufacturer’s instructions. The mitochondria were isolated through differential centrifugation and kept in a storage solution with Minute^TM^ denatured protein solution for western blotting analysis.

### Western Blotting Analysis

Total protein was extracted from rat embryonic spinal cords (E11 and E12) or C17.2 cells using radioimmunoprecipitation (RIPA) buffer (Solarbio Science & Technology, Beijing, China). Protein concentrations were determined by a bicinchoninic acid assay (Solarbio Science & Technology). Proteins were separated by 8%–12% sodium dodecyl sulfate-polyacrylamide gel electrophoresis and electrophoretically transferred to polyvinylidene difluoride (PVDF) membranes. After blocking with 5% non-fat milk solution, the membranes were incubated with primary antibodies at 4°C overnight. Following primary antibodies were applied in the experiment: anti-LC3 (Abcam, Cambridge, MA, United States; 1:1000), anti-LAMP2 (ProteinTech, Chicago, IL, United States; 1:1000), anti-Sirt1 (Cell Signaling Technology, Boston, MA, United States; 1:1000), anti-Bhlhe40 (ProteinTech, 1:1000), anti-PINK1 (ProteinTech, 1:1000), anti-VDAC1 (Abcam, 1:2000) and anti-GAPDH (ProteinTech, 1:5000). On the following day, the membranes were washed, and incubated with corresponding secondary antibodies (ProteinTech; 1:5000) at room temperature for 1.5 h. Enhanced chemiluminescent (ECL) reagent (Millipore, Billerica, MA, United States) was applied to detect protein-antibody interactions, which were visualized using a chemiluminescence detection system (C300, Azure Biosystems, United States).

### Immunofluorescence Staining

Rat embryos on E12 were collected, then fixed in 4% paraformaldehyde and paraffin embedded. Paraffin-embedded embryos were cut into 4-μm-thick sections and mounted on slides. The sections were deparaffinized in xylene, next hydrated in a graded series of ethanol. C17.2 cells were harvested and fixed with 4% paraformaldehyde, and incubated in 0.5% Triton for 30 min. The sections and cells were incubated with 5% bovine serum albumin (BSA) for 45 min in order to block non-specific sites. Then, they were incubated with normal goat serum, followed by the primary antibodies mouse anti-TOMM20 (Abcam, 1:100) and rabbit anti-LC3 (Abcam, 1:100) in a humidified chamber at 4°C overnight. After sections and cells were incubated with secondary antibodies conjugated with Alexa Fluor 488 (Cell Signaling Technology, 1:100) and Alexa Fluor 555 (Cell Signaling Technology, 1:100) were added and incubated at room temperature in the dark for 2 h. Nuclei were counterstained with 4′,6-diamidino-2-phenylindole. The specimens were observed under an ECLIPSE 80i fluorescence microscope (Nikon, Japan) or a laser scanning confocal microscopy (Ziess, Germany).

### Total RNA Isolation and Quantitative Reverse Transcription PCR (RT-qPCR)

Total RNA was extracted from rat embryonic spinal cords (from E11 to E19) or C17.2 cells using TRIzol reagent (Life Technologies, Carlsbad, CA, United States). The RNA was reverse-transcribed into cDNA using a PrimeScript RT kit (TaKaRa Biotech. Co., Ltd., China). RT-qPCR was run in an ABI 7500 Real-time PCR system (Applied Biosystems, United States) using a SYBR green master mix kit (TaKaRa). Primers for rat or mouse genes were designed and synthesized by Sangon Biotech (Sangon Biotech, Shanghai, China) (Table 1). Relative target gene expression was quantified by the 2^−ΔΔCt^ method, using *Gapdh* as an internal control.

**TABLE 1:** Sequences of the primers used for RT-qPCR analysis.

**Genes**	**Species**	**Primer**	**Sequence**
*Sirt1*	Rat	Forward Reverse	5′-TGTTTCCTGTGGGATACCTGA-3′ 5′-TGAAGAATGGTCTTGGGTCTTT-3′
*Bhlhe40*	Rat	Forward Reverse	5′-ACTGCCTATCTGCCTATGCTGGAG-3′ 5′-GGCTGCTGCTGAGGTGTTGAG-3′
*Nhlh1*	Rat	Forward Reverse	5′-ATGATGCTCAACTCCGACACCATG-3′ 5′-TCGCCCGATCCAGCACCATC-3′
*Olig3*	Rat	Forward Reverse	5′-CAAGCTCTCCAAGATCGCCACTC-3′ 5′-CTCGCCAACCAACCTCTTCATCTC-3′
*Atoh1*	Rat	Forward Reverse	5′-AACAGCGATGATGGCACAGAAGG-3′ 5′-GTGGGACCTGGGAGATGTTTTGC-3′
*Neurod1*	Rat	Forward Reverse	5′-GCCCTGGAGCCCTTCTTTGAAAG-3′ 5′-ACTCGGTGGATGGTTCGTGTTTG-3′
*Mesp2*	Rat	Forward Reverse	5′-AGAGCCTGACCAAGATCGAGACG-3′ 5′-ACTGAGGGCATCGGTGAGAGAAG-3′
*Msgn1*	Rat	Forward Reverse	5′-AAGCCTCTCCCCAGTTCCTTCTC-3′ 5′-TAGACCGCCAGCCTCGTGAC-3′
*Gapdh*	Rat	Forward Reverse	5′-GGCACAGTCAAGGCTGAGAATG-3′ 5′-ATGGTGGTGAAGACGCCAGTA-3′
*Bhlhe40*	Mouse	Forward Reverse	5′-CAGTACCTGGCGAAGCATGAGAAC-3′ 5′-TCCGAGACCACACGATGGAGATG-3′
*Sirt1*	Mouse	Forward Reverse	5′-TCCTGTTGACCGATGGACTCCTC-3′ 5′-GAGCTGGCGTGTGACGTTCTG-3′
*Gapdh*	Mouse	Forward Reverse	5′-TGTGTCCGTCGTGGATCTGA-3′ 5′-TTGCTGTTGAAGTCGCAGGAG-3′

### RNA-Seq

Total RNA was extracted from E11 and E12 rat embryonic spinal cord tissues. RNA concentrations were determined using an Agilent 2100 Bioanalyzer (Agilent RNA 6000 Nano Kit, United States). RNA purity was evaluated using a NanoDrop instrument. The BGISEQ500 platform (BGI-Shenzhen, China) was used for RNA-seq. The following 12 tissue samples were analyzed: control group of E11 (E11CON1, E11CON2, E11CON3), SBA group of E11 (E11SBA1, E11SBA2, E11SBA3), control group of E12 (E12CON1, E12CON2, E12CON3), SBA group of E12 (E12SBA1, E12SBA2, E12SBA3). Perform quality control on the raw reads to determine whether the sequencing data were fit for follow-up analysis. In total 11.30G RNA-seq data was obtained.

### Bioinformatic Analysis

In brief, differentially expressed genes (DEGs) were identified using DESeq2 (v1.4.5) with *Q*-value ≤ 0.05 (Love et al., 2014). To gain insight into DEG functions, Gene Ontology (GO) and transcription factor enrichment analyses were performed using Phyper, based on a hypergeometric test. Significantly enriched terms were Bonferroni-corrected (*Q*-value ≤ 0.05) by Bonferroni. The top 20 terms are presented.

### Luciferase Reporter Assay

Wild-type and mutant sequences of *Sirt1* mRNA sequences containing the putative Bhlhe40-binding sites were synthesized, and cloned into the pGL4.10 vector (Beijing SyngenTech). C17.2 cells were co-transfected with pcDNA3.1(+)-CMV-Bhlhe40 or pcDNA3.1(+)-CMV-MCS and the wild-type or mutant constructs. After incubating for 48 h, the C17.2 cells were harvested and subjected to a dual-luciferase reporter assay system (Promega, Madison, WI, United States) to determine binding between Sirt1 and Bhlhe40.

### Chromatin Immunoprecipitation (ChIP) Assay

ChIP assays were carried out essentially following the protocol from the SimpleChIP Enzymatic Chromatin IP Kit (Cell Signaling Technology, 9002). Briefly, C17.2 cells were treated with 1% formaldehyde for 10 min at room temperature to crosslink proteins to DNA. The chromatin was harvested and fragmented using enzymatic digestion. An aliquot of each sample was set aside as input control, while the remaining portion was subjected to immunoprecipitation with anti-Bhlhe40 antibodies (Novus Biologicals Co., Ltd., Shanghai, China) overnight at 4°C. IgG was used as a control for non-specific immunoprecipitation of DNA. Immunoprecipitated complex was treated with protease and the fragment of the Sirt1 promoter containing the E-box was amplified by RT-qPCR. The sequences of the primers were mouse Sirt1-promoter-F: 5′-GCGCCATCGCAAACTTGA-3′ and mouse Sirt1-promoter-R: 5′-CACAACACGCCGGGTCA-3′.

### Statistical Analysis

All results were analyzed with SPSS 17.0 software (SPSS Inc., Chicago, IL, United States). Data are expressed as the mean ± standard deviations (SD). Means of two groups were compared using two-tailed Student’s *t*-tests. The significant differences in *Sirt1* expression among the different treatment groups were performed by one-way analysis of variance (ANOVA) test. *p* < 0.05 was considered statistically significant.

## Results

### Mitophagy in the Spinal Cord Is Inhibited in Embryonic Rats With SBA

**FIGURE 1:**
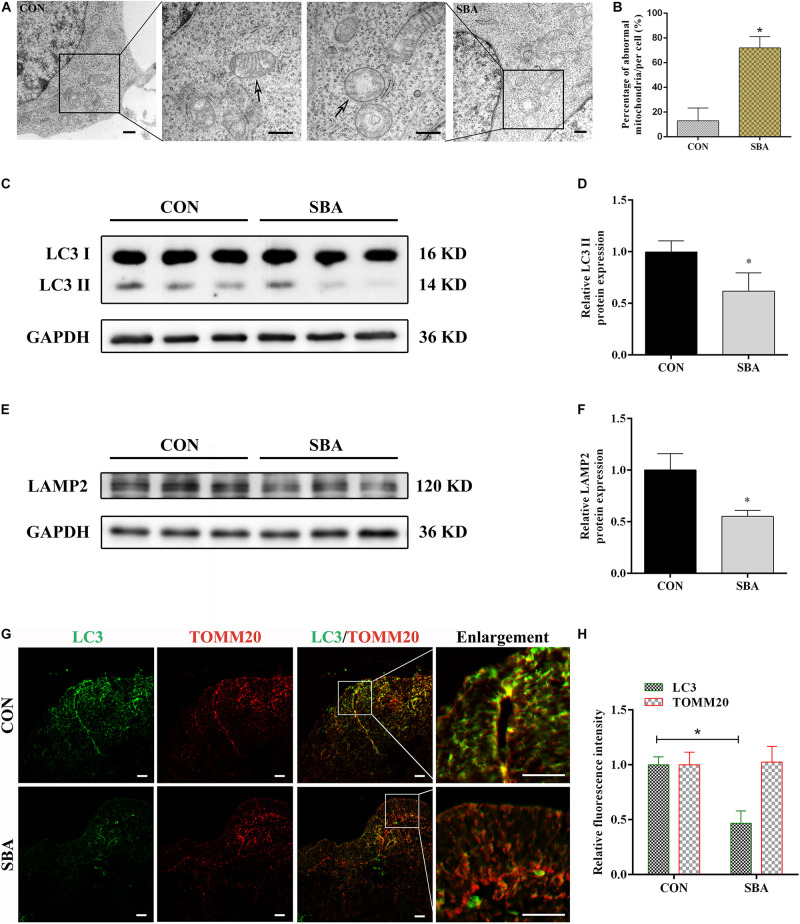
Autophagy and mitophagy in SBA rat embryos. **(A)** TEM detection of mitochondria in the neuroepithelium of spinal cords of rat embryos of the CON and SBA groups. Arrows indicate mitochondria. Scale bar = 500 nm. **(B)** Defective mitochondria rates in neuroepithelial cells from E12 embryos. Defective mitochondria rate = number of defective mitochondria divided by total number of mitochondria. **(C)** Western blot showing LC3 expression in rat embryonic spinal cord tissues in the CON and SBA groups. **(D)** Quantitation of LC3 II expression (*n* = 6). **(E)** Western blot showing LAMP2 expression in rat embryonic spinal cord tissues in the CON and SBA groups. **(F)** Quantitation of LAMP2 expression (*n* = 6). **(G)** Double immunofluorescence staining with LC3-labeled autophagosomes (green) and TOMM20-labeled mitochondria (red) in rat embryonic spinal cords. Scale bar = 10 μm. **(H)** Relative fluorescence intensities of LC3 and TOMM20 in the SBA group versus the CON group (*n* = 4). Results are expressed as the mean ± SD. *p < 0.05 vs. control group.

Mitochondrial morphology in the neuroepithelium of spinal cords of rat embryos at E12 was detected by TEM. As shown in Figure 1A, mitochondrial outer membrane wrapped the mitochondria, which were horizontally arranged in ridges. In the SBA group, the mitochondrial ridges were destroyed or even disappeared, and the matrix electron density was reduced. We found that compared to the control group, the number of defective mitochondria was significantly increased in the SBA group (Figure 1B).

Next, the expression of the autophagy marker microtubule associated protein 1 light chain 3 (LC3) and the lysosomal-specific marker lysosome-associated membrane protein 2 (LAMP2) was investigated in ATRA-induced SBA and control rats by western blotting. LC3 II expression was significantly decreased in the SBA group as compared to the CON group on E12 (Figures 1C,D). LAMP2 protein expression was notably decreased by 44.85% in the SBA group as compared to the CON group (Figures 1E,F).

To determine the occurrence of mitophagy in the ATRA-induced SBA rat model, colocalization of LC3-stained autophagosomes and translocase of outer mitochondrial membrane 20 (TOMM20)-stained mitochondria in the neuroepithelium of spinal cords was analyzed. As shown in Figures 1G,H, consistent with the western blotting results, LC3 staining was markedly weaker in the SBA group than in the CON group. TOMM20 antibody staining were relatively uniform, and no significant difference was noted between the CON and SBA group. Further, there was a lower level of LC3 puncta with TOMM20-stained mitochondria were observed in ATRA-induced SBA tissues, indicating mitophagy inhibition.

### Sirt1 Expression in the Spinal Cord Is Downregulated in Embryonic Rats With SBA

**FIGURE 2:**
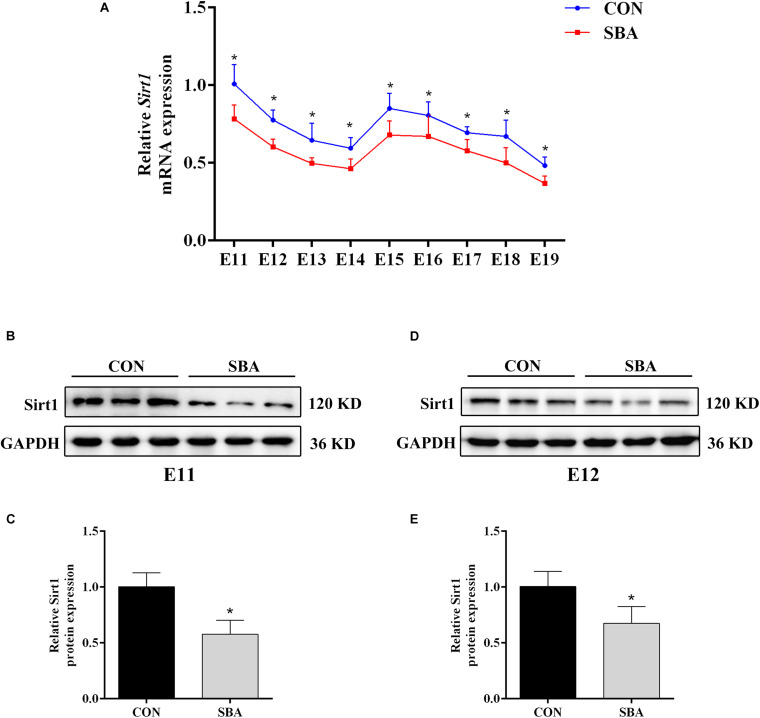
Sirt1 expression in ATRA-induced SBA. **(A)** Relative *Sirt1* mRNA expression in rat embryonic spinal cord tissues of the CON and SBA groups from E11 to E19 as assessed by RT-qPCR (*n* = 5). Results are expressed as the mean ± SD. *p < 0.05 vs. control group. **(B)** Western blot of Sirt1 in rat embryonic spinal cord tissues on E11 in the CON and SBA groups. **(C)** Quantitation of Sirt1 protein on E11 (*n* = 6). **(D)** Western blot of Sirt1 in rat embryonic spinal cord tissues on E12 in the CON and SBA groups. **(E)** Quantitation of Sirt1 protein on E12 (*n* = 6). Results are expressed as the mean ± SD. *p < 0.05 vs. control group.

Next, we investigated Sirt1 expression in the spinal cord in SBA model and control rats. RT-qPCR results indicated that *Sirt1* mRNA was decreased in the SBA group as compared to the CON group at each time point between E11 and E19 (Figure 2A). To explore whether Sirt1 protein levels were consistent with the mRNA levels, samples collected during the critical period of neural tube closure (E11 and E12) were selected for western blot analysis. Sirt1 protein was significantly downregulated in the SBA group as compared to the CON group on E11 (Figures 2B,C) and E12 (Figures 2D,E). There results suggested that Sirt1 may be involved in NTDs formation.

### RSV Increases Sirt1 and Ameliorates Autophagy Suppression *in vivo*

**FIGURE 3:**
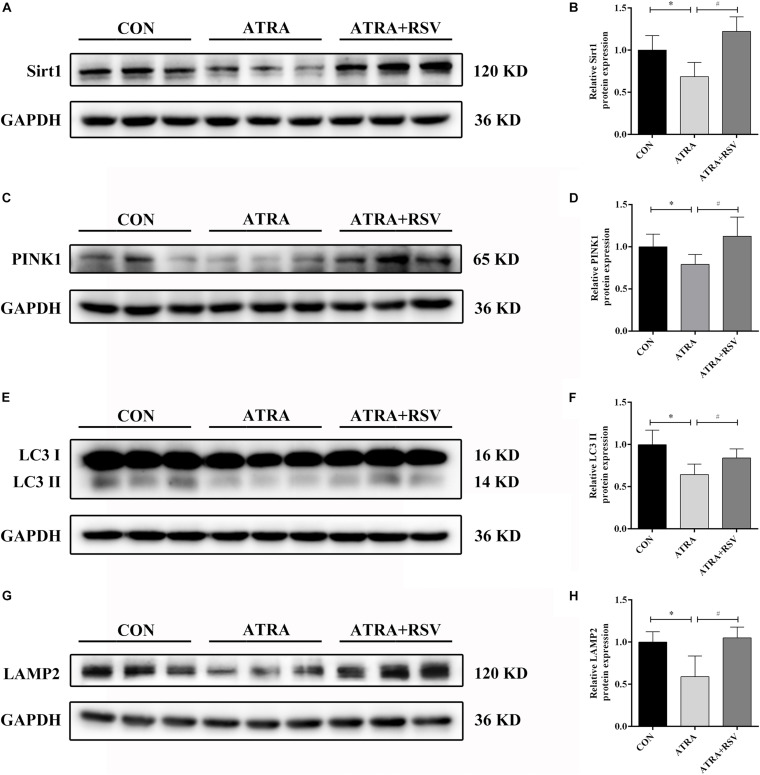
RSV activates Sirt1 and ameliorates autophagy suppression in rat embryos with ATRA-induced SBA. **(A)** Western blot of Sirt1 protein expression in rat embryonic spinal cord tissues in the CON, ATRA, and ATRA + RSV groups. **(B)** Quantitation of Sirt1 protein (*n* = 6). **(C)** Western blot of PINK1 protein expression in rat embryonic spinal cord tissues in the CON, ATRA, and ATRA + RSV groups. **(D)** Quantitation of PINK1 protein (*n* = 6). **(E)** Western blot of LC3 protein expression in rat embryonic spinal cord tissues in the CON, ATRA, and ATRA + RSV groups. **(F)** Quantitation of LC3 II protein (*n* = 6). **(G)** Western blot of LAMP2 protein in rat embryonic spinal cord tissues in the CON, ATRA, and ATRA + RSV groups. **(H)** Quantitation of LAMP2 protein (*n* = 6). The CON group tissues were taken from normal embryos, ATRA group and ATRA + SRV group tissues were taken from embryos with SBA phenotypes. Results are expressed as the mean ± SD. ^∗^*p* < 0.05 vs. CON group; ^#^*p* < 0.05 vs. ATRA group.

To confirm the function of Sirt1 in ATRA-induced SBA, pregnant dams were treated with RSV (a Sirt1 agonist) from E8 to E17. As shown in Figures 3A,B, Sirt1 expression was decreased in ATRA-treated rats as compared to CON rats on E18. Sirt1 levels in embryonic spinal cord tissues were significantly elevated after treatment with RSV. The protein level of the mitophagic marker PINK1 was also decreased in ATRA-treated rats as compared to CON rats on E18, and RSV significantly enhanced PINK1 expression as compared to ATRA treatment alone (Figures 3C,D). Further, LC3 II expression in the embryonic spinal cord tissues was significantly reduced in ATRA-treated group as compared to the CON group on E18, whereas RSV significantly increased the levels of LC3 II as compared to the ATRA treatment alone on E18 (Figures 3E,F). LAMP2 protein levels in the embryos were significantly decreased by approximately 42.98% in the ATRA-treated group as compared to the CON group on E18, whereas RSV significantly increased these levels as compared to ATRA treatment alone on E18 (Figures 3G,H).

Importantly, RSV treatment reduced the ATRA-induced SBA incidence rate in rat embryos (Table 2). These results implied that RSV treatment enhanced autophagy/mitophagy and reduced the SBA rate in ATRA-induced SBA rats.

**TABLE 2:** RSV treatment reduces the SBA rate.

	**CON**	**ATRA**	**ATRA + RSV**
Total embryos (litter)	76 (8)	86 (10)	90 (10)
SBA (%)	0	70(81.40%)	59(65.56%)
Death and resorption (%)	0	7(8.14%)	6(6.67%)

### Transcriptome Alterations in ATRA-Induced SBA Rats

**FIGURE 4:**
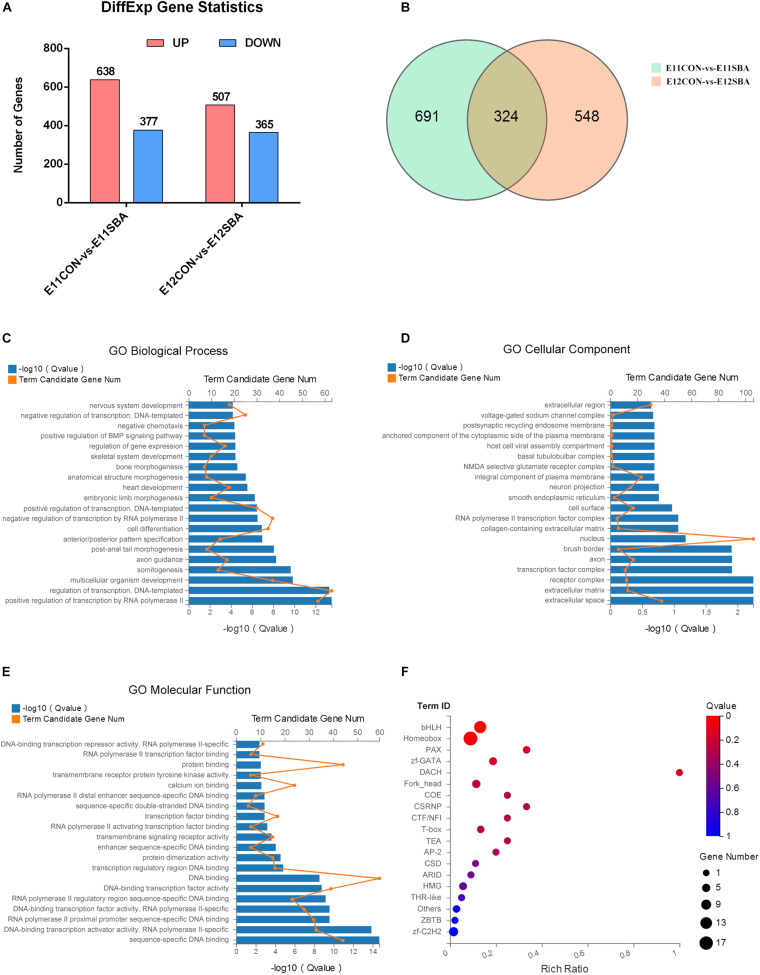
Transcriptome alterations in the ATRA-induced SBA rat model (E11 and E12). **(A)** Number of DEGs between SBA and CON rat embryos on E11 and E12. DESeq2 | log_2_FC| ≥ 0.3, *Q* ≤ 0.05. **(B)** Venn diagram of DEGs in E11CON vs. E11SBA and E12CON vs. E12SBA. **(C)** Top 20 enriched BP terms in GO analysis. **(D)** Top 20 enriched CC terms in GO analysis. **(E)** Top 20 enriched MF terms in GO analysis. **(F)** Transcription factor enrichment analysis of DEGs. Terms were considered significantly enriched when *Q* ≤ 0.05.

To explore the mechanism of NTDs and the genes regulating Sirt1 in NTDs, RNA-seq was used to identify DEGs between CON and SBA rat embryos on E11 and E12. As shown in Figure 4A, we identified DEGs, including 638 upregulated and 377 downregulated genes, in the E11SBA group compared to the E11CON group, and 872 DEGs, including 507 upregulated and 365 downregulated genes, in the E12SBA group compared to the E12CON group. The Venn diagram in Figure 4B shows that 324 DEGs were in common on E11 and E12 (Figure 4B).

Gene Ontology analysis was conducted to speculate the potential functions of those 324 DEGs. GO analysis describes genes in terms of biological process (BP) (Supplementary Table 1), cellular component (CC) (Supplementary Table 2) and molecular function (MF) (Supplementary Table 3). The top 20 GO processes ranked by enrichment score [−log_10_ (*Q*-value)] are presented. The most enriched GO terms included positive regulation of transcription by RNA polymerase II, regulation of transcription, DNA-templated, multicellular organism development and somitogenesis in BP (Figure 4C); extracellular space, extracellular matrix, receptor complex and transcription factor complex in CC (Figure 4D); sequence-specific DNA binding, DNA-binding transcription activator activity, RNA polymerase II-specific, RNA polymerase II proximal promoter sequence-specific DNA binding and DNA-binding transcription factor activity, RNA polymerase II-specific in MF (Figure 4E). Then, transcription factor enrichment analysis of the 324 DEGs further revealed that differential genes are mainly enriched in the basic helix-loop-helix (bHLH) transcription factor family was strongly enriched (Figure 4F) (Supplementary Tables 4, 5). Together, these results suggested that transcription factors are strongly involved in the regulation of ATRA-induced SBA.

### Bhlhe40 Expression Is Increased in the Spinal Cord in Embryonic Rats With SBA

**FIGURE 5:**
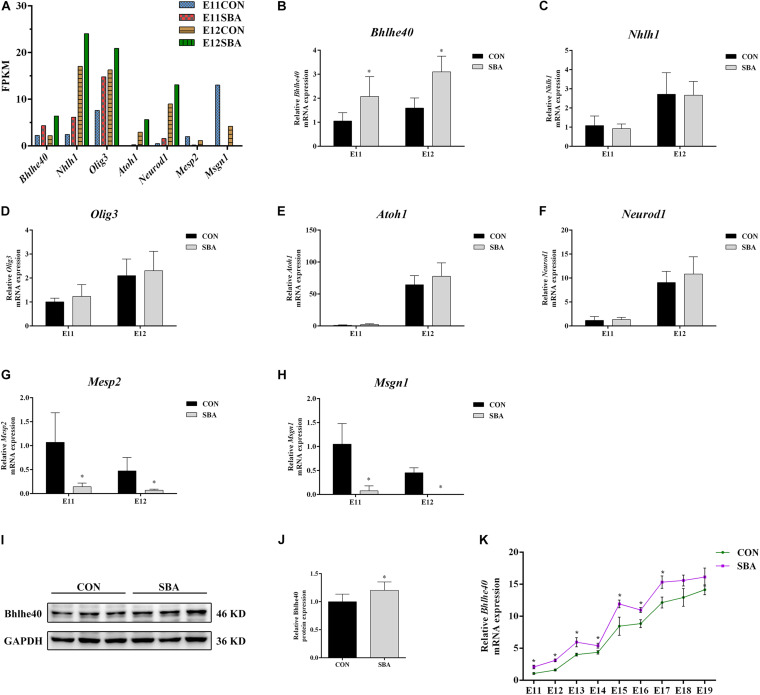
Expression of bHLH transcription factors in spinal cords of rat embryos with SBA-induced ATRA. **(A)** Average fragments per kilobase of transcript per million fragments mapped values of transcription factors in SBA rat embryos (*n* = 3). **(B–H)** Relative mRNA expression of *Bhlhe40*, *Atoh1*, *Neurod1*, *Nhlh1*, *Olig3*, *Mesp2*, and *Msgn1* in rat embryonic spinal cord tissues in the CON and SBA groups on E11 and E12 as assessed by RT-qPCR (*n* = 6). Results are expressed as the mean ± SD. ^∗^*p* < 0.05 vs. control group. **(I)** Western blot of Bhlhe40 expression. **(J)** Quantitation of Bhlhe40 expression (*n* = 6). Results are expressed as the mean ± SD. ^∗^*p* < 0.05 vs. control group. **(K)** Relative *Bhlhe40* mRNA expression in rat embryonic spinal cord tissues of the CON and SBA groups from E11 to E19 as assessed by RT-qPCR (*n* = 5). Results are expressed as the mean ± SD. ^∗^*p* < 0.05 vs. control group.

We selected bHLH transcription factor family genes that regulate neurodevelopment (*Bhlhe40*, *Atoh1*, *Neurod1*, *Nhlh1*, *Olig3*, *Mesp2* and *Msgn1*) for RT-qPCR verification. Promoter region prediction using the JASPAR database^1^ indicated that these genes harbor potential Sirt1-binding sites. The average fragments per kilobase of transcript per million fragments mapped (FPKM) values of these bHLH transcription factor genes in SBA rat embryos are presented in Figure 5A.

RT-qPCR results showed that among the upregulated genes, the mRNA level of *Bhlhe40* was increased 1.97-folds and 1.95-folds in the SBA group as compared to the CON group on E11 and E12, respectively (Figure 5B). The mRNA expression of *Mesp2* and *Msgn1* was downregulated in SBA, which was consistent with the RNA-seq results (Figures 5G,H). However, there were no significant changes in *Nhlh1*, *Olig3*, *Atoh1*, and *Neurod1* expression in the SBA group as compared to the CON group on both E11 and E12 (Figures 5C–F). As *Bhlhe40* (encoding basic helix-loop-helix family member e40) was significantly upregulated in the spinal cord in SBA embryos at an early stage and it was predicted to binding to Sirt1 with the highest, we also detected the protein expression of Bhlhe40. Compared to the CON group, protein expression of Bhlhe40 was significantly increased in the SBA group as compared to CON group (Figures 5I,J). RT-qPCR results indicated that *Bhlhe40* mRNA was significantly increased in the SBA group as compared to the CON group at each time point between E11 and E17. The mRNA level of *Bhlhe40* was increased, but has no statistically significant in the SBA group as compared to the CON group at E18 and E19 (Figure 5K).

### Bhlhe40 Binds to the Sirt1 Promoter and Suppresses Its Transcription

**FIGURE 6:**
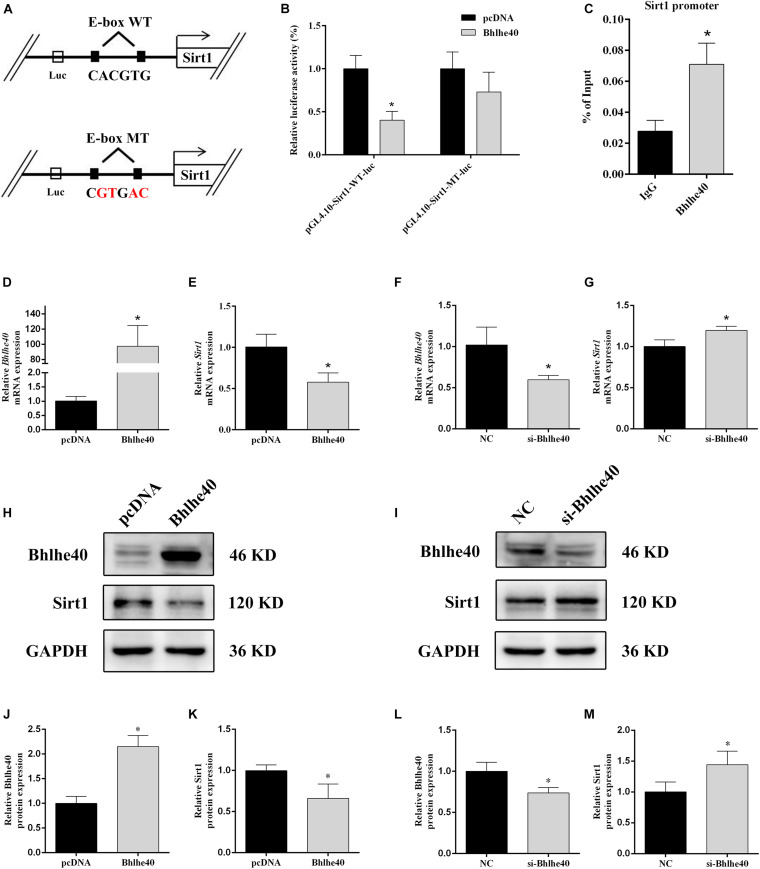
Bhlhe40 negatively regulates *Sirt1*. **(A)** Schematic representation of *Sirt1* mRNA depicting Bhlhe40-binding sites in its promoter. **(B)** Luciferase reporter assays were conducted using a *Sirt1* promoter with deleted E-box motifs after C17.2 cells were transfection with pcDNA or Bhlhe40 (*n* = 3). Results are expressed as the mean ± SD. *p < 0.05 vs. Sirt1-promoter-WT-luc + pcDNA group. **(C)** Binding of Bhlhe40 to Sirt1 promoters was examined in C17.2 by ChIP assay. Results are expressed as the mean ± SD. *p < 0.05 vs. IgG group. **(D)** RT-qPCR analysis of the *Bhlhe40* mRNA level in C17.2 cells transfected with pcDNA or Bhlhe40 (*n* = 3). **(E)** RT-qPCR analysis of the *Sirt1* mRNA level in C17.2 cells transfected with pcDNA or Bhlhe40 (*n* = 3). **(F)** Western blot analysis of Bhlhe40 and Sirt1 protein levels in C17.2 cells transfected with pcDNA or Bhlhe40. **(G)** Quantitation of Bhlhe40 (*n* = 3). **(H)** Quantitation of Sirt1 (*n* = 3). Results are expressed as the mean ± SD. *p < 0.05 vs. pcDNA group. **(I)** RT-qPCR analysis of the *Bhlhe40* mRNA level in C17.2 cells transfected with NC or si-Bhlhe40 (*n* = 3). **(J)** RT-qPCR analysis of the *Sirt1* mRNA level in C17.2 cells transfected with NC or si-Bhlhe40 (*n* = 3). **(K)** Western blot analysis of Bhlhe40 and Sirt1 protein levels in C17.2 cells transfected with NC or si-Bhlhe40. **(L)** Quantitation of Bhlhe40 (*n* = 3). **(M)** Quantitation of Sirt1 (*n* = 3). Results are expressed as the mean ± SD. ^∗^*p* < 0.05 vs. NC group.

Promoter region prediction showed that Bhlhe40 bind on the conserved E-box motifs of the *Sirt1* promoter (Figure 6A). Luciferase reporter assays confirmed binding between Sirt1 and Bhlhe40 in C17.2 cells. C17.2 cells transfected with Sirt1-promoter-WT-luc and Bhlhe40 showed significant lower luciferase activity than Sirt1-promoter-WT-luc and pcDNA transfected C17.2 cells (Figure 6B). We next performed a ChIP assay to confirm the binding of Bhlhe40 to the Sirt1 promoter in C17.2 cells. The ChIP assay results revealed that Bhlhe40 interacted with the binding sites of the Sirt1 promoter in C17.2 cells (Figure 6C).

We next transfected C17.2 cells with Bhlhe40 overexpression plasmid or Bhlhe40 siRNA to study the effect of Bhlhe40 on Sirt1. Bhlhe40 overexpression remarkably increased *Bhlhe40* mRNA (Figure 6D) and Bhlhe40 protein (Figures 6H,J) expression. Overexpressed Bhlhe40 notably decreased *Sirt1* mRNA (Figure 6E) and Sirt1 protein (Figures 6H,K) expression levels. Moreover, Bhlhe40 siRNA significantly decreased the mRNA (Figure 6F) and protein (Figures 6I,L) levels of Bhlhe40, and significantly increased the mRNA (Figure 6G) and protein (Figures 6I,M) levels of Sirt1. These findings indicated that Bhlhe40 regulates *Sirt1* transcription by directly binding to its promoter and that Bhlhe40 and Sirt1 expression is negatively correlated.

### The Bhlhe40/Sirt1 Axis Regulates Mitophagy *in vitro*

**FIGURE 7:**
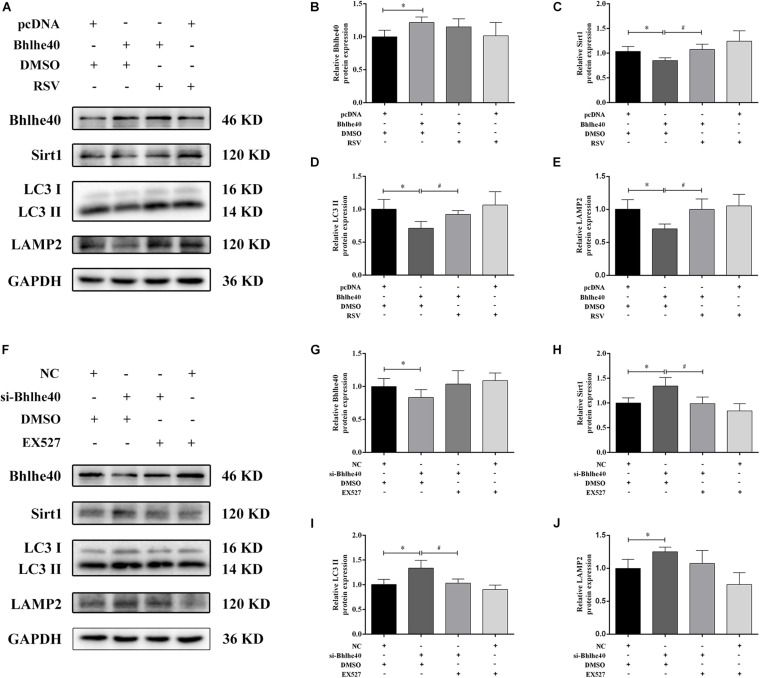
The Bhlhe40/Sirt1 axis regulates autophagy/mitophagy-related proteins in C17.2 cells. **(A)** Western blot analysis of Bhlhe40, Sirt1, LC3, and LAMP2 protein levels in C17.2 cells treated with different combinations of pcDNA, Bhlhe40, DMSO, and RSV. Quantitation of Bhlhe40 **(B)**, Sirt1 **(C)**, LC3 II **(D)**, and LAMP2 **(E)** protein (*n* = 3). Results are expressed as the mean ± SD. ^∗^*p* < 0.05 vs. pcDNA + DMSO group; ^#^*p* < 0.05 vs. Bhlhe40 + DMSO group. **(F)** Western blot analysis of Bhlhe40, Sirt1, LC3 II, and LAMP2 protein in C17.2 cells treated with different combinations of NC, si-Bhlhe40, DMSO, and EX527. Quantitation of Bhlhe40 **(G)**, Sirt1 **(H)**, LC3 **(I)**, and LAMP2 **(J)** protein (*n* = 3). Results are expressed as the mean ± SD. ^∗^*p* < 0.05 vs. NC + DMSO group; ^#^*p* < 0.05 vs. si-Bhlhe40 + DMSO group.

To investigate the regulatory role of the Bhlhe40/Sirt1 axis in mitophagy function, we used western blotting to examine autophagy-related proteins and double immunofluorescence staining to detect mitophagy in C17.2 cells. Bhlhe40 overexpression significantly decreased the protein levels of Sirt1 (Figures 7A–C), the autophagy marker LC3 II (Figures 7A,D), and the lysosomal-specific marker LAMP2 (Figures 7A,E), which were rescued by RSV treatment. Inversely, Bhlhe40 siRNA significantly increased the protein levels of Sirt1 (Figures 7F–H), LC3 II (Figures 7F,I), and LAMP2 (Figures 7F,J), which were abolished by the Sirt1 inhibitor EX527.

**FIGURE 8:**
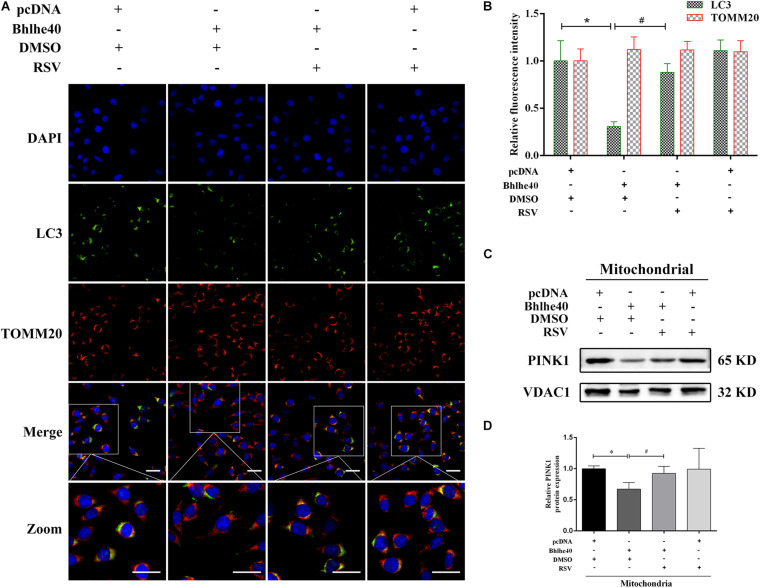
The Sirt1 activator RSV attenuated mitophagy inhibition caused by Bhlhe40 in C17.2 cells. **(A)** C17.2 cells were double immunofluorescence-stained, with LC3-labeled autophagosomes (green) and TOMM20-labeled mitochondria (red). Scale bar = 25 μm. **(B)** Relative fluorescence intensities of LC3 and TOMM20 in each group (*n* = 3). Results are expressed as the mean ± SD. *p < 0.05 vs. pcDNA + DMSO group; ^#^ *p* < 0.05 vs. Bhlhe40 + DMSO group. **(C)** Western blot analysis of PINK1 in mitochondria isolated from C17.2 cells treated with different combinations of pcDNA, Bhlhe40, DMSO, and RSV. **(D)** Quantitation of PINK1 protein (*n* = 3). Results are expressed as the mean ± SD. ^∗^*p* < 0.05 vs. pcDNA + DMSO group; ^#^*p* < 0.05 vs. Bhlhe40 + DMSO group.

Consistent with the western blotting results, LC3 staining was weakened in the Bhlhe40 overexpression group compared with the pcDNA group, whereas it was increased by RSV treatment (Figures 8A,B). Further, we observed a lower level of colocalization of stained LC3 puncta with TOMM20-stained mitochondria, indicating mitophagy inhibition. The Sirt1 activator RSV attenuated mitophagy inhibition caused by Bhlhe40 in C17.2 cells. PINK1 is characterized by expressing on damaged mitochondria, which regulates Parkin trafficking to phagosomes and drives dysfunctional mitochondria removal via mitophagy (Gelmetti et al., 2017). Western blotting analysis also showed PINK1 was decreased by Bhlhe40 overexpression, whereas increased after RSV treatment in mitochondria of C17.2 (Figures 8C,D). Together, these data implied that Bhlhe40 regulates mitophagy in C17.2 cells by targeting Sirt1.

## Discussion

Despite advances in the elucidation of the pathogenesis and in the treatment of NTDs, current treatments are still limited and unsatisfactory. In this study, we made three major findings. First, we observed mitophagy impairment in ATRA-induced rat embryonic SBA. Second, administration of RSV activated Sirt1 to attenuate autophagy suppression and ameliorate SBA *in vivo*. Third, we demonstrated for the first time that the Bhlhe40/Sirt1 axis regulates mitophagy function *in vitro*.

The maintenance of a healthy and functioning mitochondrial network is critical during development (Pickles et al., 2018). Mitochondria are complex organelles that serve as the cellular powerhouse and are involved in a wide range of key cellular functions, including redox signaling, and the regulation of metabolic homeostasis, cells survival, and cell death (Lou et al., 2020). Emerging evidence suggests that mitochondria are critical regulatory organelles in determining neural stem cell fate, both in initial neural development and in adult neurogenesis (Khacho et al., 2019). Our previous study have showed that mitochondrial respiratory efficiency, adenosine triphosphate content, as well as antioxidant enzyme activity were decreased in spinal cords of early embryos with SBA, which may have caused high ROS levels, leading to mitochondrial dysfunction (Xue et al., 2018). In this study, we observed mitochondrial crest blurring and mitochondrial swelling in early embryos with SBA by TEM, which suggested that damaged mitochondria may not be effectively eliminated.

Autophagy, a lysosome dependent pathway that leads to the degradation of dysfunctional organelles and proteins, plays a crucial role in early embryonic development (Tsukamoto et al., 2008). LC3 is a soluble protein widely distributed in mammalian tissues as well as in cultured cells. LC3 II is formed by conjugation of LC3 I to phosphatidylethanolamine and is defined as a marker of autophagosomes (Tanida et al., 2008). In the present study, LC3 II was observed to be downregulated in the spinal cord in ATRA-induced SBA rats compared with control rats. We also detected the lysosomal-specific marker LAMP2, as lysosomes play a key degrading role during autophagy (Zhou et al., 2013). LAMP2 expression was significantly decreased in the spinal cords of ATRA-induced SBA rats compared with control rats, which suggested that autophagy was inhibited. In addition to maintaining cellular homeostasis, autophagy eliminates damaged organelles for cellular quality control purposes. Notably, damaged mitochondria can be eliminated through the mitophagy pathway to maintain mitochondrial homeostasis, energy production, and neuronal survival (Wang and Wang, 2019; Wang et al., 2020). In other words, mitophagy dysfunction can result in the accumulation of damaged mitochondria, which in turn is associated with a various neurological pathological conditions (Yang et al., 2018). To investigate whether mitophagy is involved in the pathogenesis of NTDs, we examined the expression of double-labeling for LC3 and TOMM20 by double-labeling immunofluorescence analysis. TOMM20 plays an essential role as a receptor for proteins targeted to mitochondria (Park et al., 2019). In our study, results showed the integration of autophagosomes and damaged mitochondria was inhibited in the SBA group, which suggested that mitophagy was blocked. These results implied that mitophagy was inhibited, which may be the reason of mitochondrial dysfunction in the spinal cords of embryonic rats with SBA.

Sirt1 is important for the turnover of defective mitochondria by mitophagy, and it upregulates autophagy/mitophagy through multiple known pathways (Tang, 2016; Fang, 2019). Mice lacking the gene *Sirt1* were small, exhibited notable developmental defects of the retina and heart, and died a few hours after birth (Cheng et al., 2003; Wang et al., 2008; Zhang et al., 2020), suggesting that Sirt1 plays a vital role in mouse embryonic stem cell maintenance and embryonic development (Tang et al., 2017). Indeed, Sirt1 plays protective roles in some neurodegenerative diseases, including motor neuron diseases, and Parkinson’s disease (Herskovits and Guarente, 2014). In our study, Sirt1 expression was significantly decreased in ATRA-induced SBA compared to normal rat embryonic spinal cord tissues. RSV, a natural plant polyphenol, has also been identified as a Sirt1 activator, and a number of studies have linked the beneficial effects of RSV to modulation of Sirt1’s activity (Galiniak et al., 2019). Further, RSV has shown neural protective effects of RSV has been shown in the central nervous system in experimental models (Jardim et al., 2018). In addition, RSV protected against oxidative stress and cell apoptosis in embryos in diabetic embryopathy model rats (Singh et al., 2011). In our rat model, we found RSV attenuated autophagy/mitophagy-associated proteins (Sirt1, PINK1, LC3 II and LAMP2) suppression by ATRA-induced SBA. Moreover, RSV administration reduced the incidence rate of SBA. These data indicated that RSV centrally activates Sirt1, enhancing autophagy/mitophagy and improving the embryonic outcome.

To explore the molecular mechanisms underlying mitophagy in NTDs, further, we compared fetal spinal cord RNA profiles of SBA embryos and normal embryos in the early embryonic stage using RNA-seq. We identified 324 DEGs between the CON and ATRA-induced SBA groups on both E11 and E12. Importantly, GO BP and MF term analysis showed that transcription factors were strongly enriched among the DEGs. Moreover, transcription factor enrichment analysis of the DEGs indicated that the bHLH transcription factor family was significantly enriched. Transcription factors containing the bHLH domain participate in several stages of neurodevelopment, including neurite growth and neuron differentiation (Kabos et al., 2002). Among the candidate DEGs, *Bhlhe40* was selected for further study because it was significantly upregulated in the spinal cords of SBA embryos at an early stage and it was predicted to binding to Sirt1 with the highest.

Bhlhe40 is extensively and strongly expressed in nervous tissues (Shi et al., 2010). As a transcriptional repressor, Bhlhe40 binds to target gene promoters to suppress gene expression. Bhlhe40 regulates different targets in different organs, and may have differential roles in various diseases. For example, Bhlhe40 facilitates PI3K/Akt/mTOR activation and triggers tumor progression in breast cancer, whereas it represses STAT1 expression and activates tumor suppression in clear cell carcinoma (Kiss et al., 2020). Downregulation of Bhlhe40 in the prefrontal cortex of spontaneously hypertensive rats improved their hyperactive behavior. Overexpressed Bhlhe40 inhibited the differentiation of PC12 cells *in vitro*, whereas Bhlhe40 silencing promoted neurite axon and dendrite outgrowth (Wu et al., 2017). Remarkably, we found that Bhlhe40 was significantly upregulated in ATRA-induced SBA rat embryonic spinal cords. This suggested that Bhlhe40 may play a regulatory role in the onset of NTDs. We identified two possible Bhlhe40-response elements (consensus E-box: CACGTG) in the promoter region of Sirt1. As expected, forced expression of Bhlhe40 significantly repressed *Sirt1* promoter activity. Importantly, Bhlhe40 overexpression repressed Sirt1 mRNA and protein expression, and Bhlhe40 siRNA significantly enhanced Sirt1 mRNA and protein expression. Taken together, these results suggest that Bhlhe40 negatively regulates *Sirt1* transcription by directly binding to its promoter.

As a transcriptional inhibitor, Bhlhe40 is involved various cellular events, including cell proliferation and differentiation, apoptosis, neurogenesis and tumorigenesis (Kabos et al., 2002; Zheng et al., 2009; Kiss et al., 2020). However, the potential role of Bhlhe40 in autophagy/mitophagy had not been elucidated. In our study, overexpressed Bhlhe40 significantly decreased the expression of PINK1, LC3 II and LAMP2, and LC3 puncta colocalized less with TOMM20-stained mitochondria in Bhlhe40-overexpressing cells, indicating mitophagy inhibition. RSV is capable of stimulates autophagy/mitophagy by activating Sirt1, and therefore has shown great potential in the treatment of several diseases. Pre-treatment with RSV reduced the benzo (a) pyrene-induced mitochondrial dysfunction and apoptosis via mitophagy regulation in neurons (Kang et al., 2020). In support of this, our study showed that RSV treatment strongly ameliorated Bhlhe40-induced LC3 II and LAMP2 inhibition in C17.2 cells. Consistent herewith, the Sirt1 inhibitor EX527 (Chen G. et al., 2017), reversed the effects of Bhlhe40 siRNA on LC3 II and LAMP2 expression. PINK1-mediated mitophagy is currently the most well-established way mediate mitophagy in mammalian cells (Youle and Narendra, 2011). Depletion of PINK1, a key mitophagic regulator, impaired mitophagy and blocked the protective role of Sirt1 against compression induced senescence in nucleus pulposus cells (Wang et al., 2020). In our study, we showed that increased level of PINK1 protein after Sirt1 activator RSV treatment in isolated mitochondrial protein. Moreover, suppression of mitophagosomes formation by Bhlhe40 was rescued by RSV in C17.2 cells. Together, these data suggested that the Sirt1/Bhlhe40 axis regulates mitophagy. Further studies on the function of the Bhlhe40/Sirt1 axis *in vivo* are needed.

In summary, the present study provided evidence of abnormal mitophagy and differential Sirt1 expression in a rat model of ATRA-induced SBA. Moreover, we demonstrated that maternal RSV treatment activated Sirt1 to attenuate autophagy suppression and ameliorate SBA *in vivo*. Transcriptome analysis showed that transcriptional regulation plays an important role in NTDs. Furthermore, we found that the Bhlhe40/Sirt1 axis regulates mitophagy in neural stem cells. Our findings provide new insights in the pathogenesis of NTDs and may pave the way for potential therapeutic targets for NTDs.

## Data Availability Statement

The original contributions presented in the study are included in the article/Supplementary Material, further inquiries can be directed to the corresponding author.

## Ethics Statement

The animal study was reviewed and approved by the Animal Ethics Committee of Shengjing Hospital of China Medical University.

## Author Contributions

ZY and LZ contributed to the study concepts. LZ contributed to the experiment performance. LZ, DL, WM, HG, XW, and WL contributed to the data analysis. LZ wrote the manuscript. ZY made manuscript revisions. All authors contributed to the article and approved the submitted version.

## Conflict of Interest

The authors declare that the research was conducted in the absence of any commercial or financial relationships that could be construed as a potential conflict of interest.

## Footnotes

1 http://jaspar.genereg.net/
